# Virtual Coach–Guided Online Acceptance and Commitment Therapy for Chronic Pain: Pilot Feasibility Randomized Controlled Trial

**DOI:** 10.2196/56437

**Published:** 2024-11-08

**Authors:** Erin D Reilly, Megan M Kelly, Hannah L Grigorian, Molly E Waring, Karen S Quigley, Timothy P Hogan, Alicia A Heapy, Charles E Drebing, Matias Volonte, Ummul-Kiram Kathawalla, Hannah E Robins, Katarina Bernice, Timothy Bickmore

**Affiliations:** 1 Mental Illness Research, Education, and Clinical Center Veteran Affairs Bedford Healthcare System Department of Veteran Affairs Bedford, MA United States; 2 Department of Psychiatry University of Massachusetts Chan Medical School Worcester, MA United States; 3 Department of Allied Health Sciences University of Connecticut Storrs, CT United States; 4 College of Science Northeastern University Boston, MA United States; 5 Center for Healthcare Organization and Implementation Research Veterans Affairs Bedford Healthcare System Department of Veterans Affairs Bedford, MA United States; 6 Peter O'Donnell Jr School of Public Health University of Texas Southwestern Medical Center Dallas, TX United States; 7 Pain Research, Informatics, Multi-morbidities, and Education Center Veterans Affairs Connecticut Healthcare System Department of Veterans Affairs West Haven, CT United States; 8 School of Medicine Yale University New Haven, CT United States; 9 Cheyenne Veterans Affairs Medical Center Department of Veterans Affairs Cheyenne, WY United States; 10 School of Computing Clemson University Charleston, SC United States; 11 Department of Clinical Psychology Suffolk University Boston, MA United States; 12 Khoury College of Computer Sciences Northeastern University Boston, MA United States

**Keywords:** chronic pain, randomized controlled trial, usability, acceptance and commitment therapy, embodied conversational agent, veterans

## Abstract

**Background:**

Veterans are disproportionately affected by chronic pain, with high rates of pain diagnoses (47%-56%) and a 40% higher rate of prevalence of severe pain than nonveterans. This is often accompanied by negative functional outcomes and higher mortality. Combined with research suggesting medical treatments for chronic pain are often insufficient, there is an urgent need for nonmedical pain self-management programs. An interactive online platform to deliver an efficacious treatment for chronic pain such as acceptance and commitment therapy (ACT) could be a valuable option to assist veterans with pain care at home.

**Objective:**

This study aims to evaluate the virtual coach–guided Veteran ACT for Chronic Pain (VACT-CP) online program compared to a waitlist and treatment as usual (WL+TAU) control group through a small pilot feasibility randomized controlled trial. The primary aim was to evaluate the feasibility and acceptability of VACT-CP and study procedures, such as ease of recruitment, treatment receptivity, attrition and retention, sustained participation, system usability, and assessment of trial procedures. Secondary aims explored differences in the VACT-CP and WL+TAU groups on pre- and posttest (week 7) outcome measures for pain, mental health, functioning, and ACT processes.

**Methods:**

Veterans with chronic pain were recruited and randomized to either the VACT-CP (n=20) or the WL+TAU (n=22) group in a parallel group trial design. Self-report surveys were administered to participants at baseline (week 0), at the intervention midpoint (week 3), immediately after the intervention (week 7), and at the 1-month follow-up (week 11). We used Wilcoxon signed rank tests with the intention-to-treat sample to describe changes in secondary outcomes from pre- to postintervention within each group.

**Results:**

Study procedures showed good feasibility related to recruitment, enrollment, randomization, and study completion rates. Participants reported that VACT-CP was easy to use (System Usability Scale: mean 79.6, SD 12.8; median 82.5, IQR 70-87.5); they completed an average of 5 of the 7 total VACT-CP modules with high postintervention satisfaction rates. Qualitative feedback suggested a positive response to program usability, content tailoring, veteran centeredness, and perceived impact on pain management. Although the pilot feasibility trial was not powered to detect differences in clinical outcomes and significant findings should be interpreted with caution, the VACT-CP group experienced significant increases in chronic pain acceptance (*P*<.001) and decreases in depressive symptoms (*P*=.03).

**Conclusions:**

VACT-CP showed encouraging evidence of feasibility, usability, and acceptance, while also providing promising initial results in improving a key process in ACT for chronic pain—chronic pain acceptance—after online program use. A full-scale efficacy trial is needed to assess changes in clinical outcomes.

**Trial Registration:**

ClinicalTrials.gov NCT03655132; http://clinicaltrials.gov/ct2/show/NCT03655132

**International Registered Report Identifier (IRRID):**

RR2-10.2196/45887

## Introduction

### Background

Pain is one of the most common medical concerns reported by US veterans, particularly in primary care settings [1]. Perhaps the most complicated and devastating condition to treat, chronic pain refers to self-reported unpleasant sensory and emotional experiences associated with, or resembling that associated with, actual or potential tissue damage that persists and recurs for >3 months, often lasting for years [2]. Chronic pain is a highly prevalent problem, with an estimated 126.1 million US adults reporting some pain in the previous 3 months and 50 million adults (20.4%) reporting daily chronic pain [3]. Veterans also present with disproportionately high rates of chronic pain (47%-56%) and with a 40% higher prevalence of severe pain than nonveterans [1]. In an effort to reduce pain impacts, pharmacological treatments such as long-term opioid therapy are often prescribed [4]. While short-term use of opioids can provide some relief for patients with chronic pain, traditional medical approaches raise significant concerns for long-term use [5]. In fact, there is no evidence of long-term efficacy of prescription opioids in chronic pain treatment, which can lead to problematic substance use and death [6-8]. These findings have led to the recent US Centers for Disease Control and Prevention clinical guidelines promoting nonpharmacological interventions, in particular, psychosocial interventions, as a first-line treatment [9]. To more safely address pain in patients, it is crucial to look beyond pharmacological treatments and consider not only the physical aspects of chronic pain but also the cognitive and emotional factors involved.

Because the best modalities for chronic pain treatment emphasize the relationship between pain, physical illness, and emotional distress, veteran health researchers suggest that treatment for chronic pain be multimodal, multidisciplinary, and transdiagnostic [10-12]. This biopsychosocial approach to chronic pain acknowledges the importance of the pain cycle in the chronification and maintenance of pain [13]. Specifically, this refers to the process by which primary physical pain first leads to physical issues and sensations such as muscles spasms, guarding, and inflammation. This, in turn, can prompt individuals to restrict their mobility and activities, leading to muscle weakness and loss of functioning. Consequently, this can result in strong feelings of anger, frustration, and hopelessness in addition to increased or continued pain. The pain cycle emphasizes that although rest and avoidance of physical, social, and recreational demands may be clinically indicated following the initial occurrence of pain, continued use of this approach actually increases the intensity and negative impact of chronic pain over time [14]. In alignment with the fear-avoidance model of chronic pain, this continues as pain is catastrophized, behavior is avoided, and a vicious cycle is maintained by fearful hypervigilance [15]. This cycle also highlights the importance of addressing not just pain reduction but avoidance of and barriers to activity engagement. Consequently, behavioral approaches that target issues related to the pain-catastrophizing cognitions, emotional concerns, and engagement in meaningful activities related to chronic pain have been well documented for reduction of associated psychiatric symptoms and modest pain relief [16].

With >20 years of empirical research supporting its efficacy, acceptance and commitment therapy (ACT) encapsulates the strengths of several existing chronic pain treatment approaches for improved functioning in veterans by targeting the biopsychosocial nature of pain. ACT focuses on the identification of a person’s valued life goals, the acceptance of difficult internal and external experiences, and commitment to actions that are consistent with one’s values [17]. This often includes directly noticing and addressing painful emotional experiences and catastrophizing thoughts that increased physical activity or engagement in valued life activities will lead to the reoccurrence or intensification of pain. By supporting individuals in breaking their “fusion” with pain-catastrophizing thoughts and learning strategies to engage with valued life activities even in the context of ongoing pain, ACT is able to directly address major issues in the fear-avoidance model of chronic pain that contribute to poor functioning and quality of life [18]. ACT has been shown to be particularly effective in improving functioning and quality of life in the context of managing chronic pain [19]. A systematic review of ACT for chronic pain intervention studies suggests that ACT is efficacious for enhancing general and physical functioning and lowering pain-related distress [20]. ACT interventions aim to increase acceptance of chronic pain in the present moment, which leads to more flexibility in one’s response to pain and its management; multiple effectiveness studies investigating this have produced effect sizes in the medium to large range across different clinical outcome domains, showing positive impacts on social functioning and decreased pain-related medical visits even 3 years after treatment [20].

Behavioral treatments for chronic pain can be labor intensive and less accessible to veterans outside of major Veterans Affairs (VA) settings, with less access to therapists trained in chronic pain treatment [21,22]. Fewer than half of the veterans with chronic pain receive psychosocial treatment (eg, mental health therapy and support groups) for managing chronic pain, and although expanding, many VA care facilities do not have any pain-focused psychological services that use behavioral therapeutic orientations [21]. Online interventions for psychiatric and behavioral issues have been gaining popularity [23,24], as 91% of adults have access to the internet at home or use wireless mobile devices [25]. This suggests that the VA could benefit from the rapidly expanding field of online and mobile health technology for increased access to empirically supported chronic pain treatments.

Outside of the VA, the use of online-delivered ACT for chronic pain has been rapidly expanding and found to increase activity engagement, pain acceptance, sleep quality, and quality of life, while decreasing pain-related distress, anxiety, and depressive symptoms [26,27]. In addition, meta-analytic analyses to determine the efficacy of online ACT for adults with chronic pain compared with controls has found that online ACT can result in medium effects on pain interference and pain acceptance and small effects on pain intensity, depression, anxiety, mindfulness, and psychological flexibility on postintervention follow-ups [28]. Despite multiple additional studies on civilian populations suggesting that online-delivered ACT can improve chronic pain management [29,30], targeted web-based ACT therapy interventions for veterans have not been developed or investigated. This is unfortunate, given there is research suggesting that veterans with a range of mental and physical health conditions are interested in mobile interventions [31]. In addition, although digital health programs are becoming increasingly popular and efficacious, sustained engagement in online interventions tends to be poor for these at-home treatments [32].

To both guide mobile interventions and address difficulties with engagement, online-supported mental health interventions have begun to explore the use of animated characters, also known as embodied conversational agents (ECAs), as personal virtual guides to support behavioral interventions [33]. Studies suggest that using ECAs can boost motivation and provide valuable feedback in online treatments, leading to improved treatment adherence, increased physical activity and diet compliance, and greater achievement of client goals [21]. The use of ECAs has been increasingly adopted to guide psychotherapeutic cognitive and behavioral interventions as well [34]. However, although the research is promising, the impact and perception of ECAs as guides for therapeutic treatment, especially in veteran populations, is not yet clear. Therefore, it is important to gather more feedback and evidence regarding the usability, feasibility, and acceptability of these online pain management programs for veterans with chronic pain. This approach will help developers, providers, and clinical systems better understand how to design and implement technology that meets the care needs of veterans with chronic pain in a home setting.

### Objectives

Our collaborative, interdisciplinary research group created an online ECA-delivered Veteran ACT for Chronic Pain (VACT-CP) program to support veterans in managing their pain and improving their mental and physical health functioning (pilot trial protocol paper available [35]). Following initial development and usability testing of the intervention and consistent with the National Institutes of Health–recommended stage model for development of behavioral therapies [36], we conducted a pilot feasibility randomized controlled trial (RCT) to assess the feasibility and acceptability of the VACT-CP (n=20) program compared to a waitlist and treatment as usual (WL+TAU; n=22) control group. We examined the following study aims:

- The primary aim was to assess the feasibility, usability, and acceptability of VACT-CP and a WL+TAU comparison condition, including ease of recruitment, attrition and retention in each group, sustained VACT-CP website use, treatment receptivity, and the assessment process.
- The secondary aim was to describe changes in pain acceptance, valued living, mental and physical functioning, pain-related interference in daily functioning, and behavioral avoidance among participants in each treatment group.

## Methods

### Ethical Considerations

This study was approved by the institutional review board of the VA Bedford Healthcare System, Bedford, Massachusetts (#1598754-11), and the requirement for written informed consent was waived. Data were deidentified. Participants were compensated US $60 for the baseline assessment, US $40 at the midpoint testing visit at week 3 for the WL+TAU group, US $60 for the end of treatment (session 7) at the end of week 7 for the WL+TAU group, and US $40 for the 1-month follow-up assessment (total possible compensation of US $200 in either condition). Participants were compensated immediately after completing each assessment time point, and all compensation was provided via gift cards for Walmart, Amazon, or CVS Pharmacy, depending on participant preference.

### Study Trial Design

The aim of this research study was to conduct a stage IB pilot feasibility RCT to assess the usability, feasibility, and acceptability of the VACT-CP program compared with the outcomes in the WL+TAU control group. Participants were recruited only in 1 group arm; thus a parallel group trial design was used with a 1:1 allocation ratio. After participating in the study, those in the WL+TAU arm were given the opportunity to use the VACT-CP website program if they desired, but these participants were no longer tracked or followed at that point. Consequently, no additional study intervention was administered by staff in the WL+TAU control arm in this design. No changes were made to the planned methods after the pilot commenced.

### Participants

Participants were recruited over the course of 10 months and screened for the presence of chronic pain and other inclusion and exclusion criteria. First, potential participants were prescreened by telephone to determine whether they met the basic study eligibility criteria before scheduling a baseline appointment. Inclusion criteria included the following: (1) was a veteran aged ≥18 years; (2) had a current diagnosis of noncancer chronic pain, defined as having at least 1 pain-related diagnosis indicated by an *International Classification of Diseases, Ninth Revision* or *International Classification of Diseases, Tenth Revision* code related to either musculoskeletal pain or joint problems or osteoarthritis, as defined in the veteran’s existing Veterans Health Administration (VHA) medical record and presence of either chronic pain of at least mild to moderate severity as indicated by ≥2 numerical rating scale (NRS) pain scores of ≥4 at 2 separate VA outpatient visits in the past year based on a VA medical record review or a grade 1 or 2 indication on the Graded Chronic Pain Scale if VA medical note with NRS pain scores were not present in the VA medical record [37]; (3) had a working, high-speed wireless internet connection at home or was willing to access the website over the 7-week intervention at the VA Bedford Healthcare System using a provided laptop computer in a secure space; and (4) was competent to provide written informed consent.

Participants were excluded if they met any of the following criteria: (1) had a current or lifetime diagnosis of a psychotic disorder as per the *Diagnostic and Statistical Manual of Mental Disorders, Fifth Edition* (*DSM-5*); (2) had a current or recent (within 1 month of study entry) *DSM-5* alcohol or drug use disorder; (3) was undergoing any other chronic pain-related behavioral or psychological treatment; (4) had any cognitive impairment that would interfere with study participation; (5) presented clinically significant suicidality within the past year; (6) presented any clinical features requiring a higher level of care (eg, inpatient treatment); and (7) had any cognitive or physical impairment that would interfere with aspects of study participation that require using a computer and providing feedback. During the study, an additional study criterion was added by the principal investigator (PI) to exclude participants who had participated in previous website usability testing for VACT-CP, as they had already completed portions of modules 1 and 2 of the VACT-CP program and, thus, had received at least part of the VACT-CP treatment.

Veterans were recruited via the VA Bedford Healthcare System (Bedford, Massachusetts) using hospital-posted flyers, presentations to clinical care services (eg, social work services), community outreach (ie, having staff present at study recruitment table with flyers and to answer questions during veteran-specific and VHA-sponsored events in the Massachusetts community), provider referrals, and recruitment letters. Research staff prescreened interested veterans telephonically and reviewed eligible individual’s medical records to confirm study eligibility. Participants were asked to complete study surveys (available either online or using paper and pencil) at 4 time points over the course of their 11-week study participation (baseline or week 0, midpoint or week 3, postintervention or week 7, and 1-month follow-up or week 11).

Individuals who were deemed eligible after the telephonic prescreening had their existing VA medical records reviewed by study staff to assess whether additional inclusion or exclusion criteria were met. This included reviewing for the existence of a noncancer chronic pain diagnosis, lifetime psychotic disorder, or recent (within 1 month) substance use disorder (SUD) in the veteran’s medical record, all of which were diagnosed by a licensed clinical health provider with the VA health care system. For veterans found eligible after this screening process, a baseline appointment was scheduled, either online or in person, to complete the informed consent process and baseline measures. The informed consent process involved study staff and the potential participant verbally going through the study information sheet, and after this review, participants verbally provided consent to participate in study activities. In addition, the following measures were implemented after obtaining consent: (1) a mental status exam to assess whether a participant was oriented to time, place, and person; (2) a structured clinical interview for *DSM-5* [38] completed by the PI or trained research staff to assess for exclusion criteria related to potential psychosis, SUDs, and suicidality that may not have been present in the medical record; and (3) the baseline survey battery, which was administered via either the Qualtrics (Qualtrics, LLC) online survey platform or paper and pencil, depending on the participant’s preference. Participants were also provided with a study orientation, which included a brief PowerPoint (Microsoft Corp) presentation that outlined the study rationale, participant activities, study timeline, and the importance of providing follow-up data for the study regardless of one’s group assignment, consistent with the best practices for enhancing clinical trial retention [39,40]. Eligible veterans who confirmed their continued interest in and consent to participating in the study were then randomized by the study staff to either the VACT-CP group or the WL+TAU control group. Due to higher-than-anticipated interest and eligibility of veterans during the screening process, a fifth randomization block was created, and 2 additional eligible participants were randomized within this block instead of denying them the opportunity to participate after reaching the original goal of 40 participants.

### Interventions

#### VACT-CP Online Intervention Group

Participants in the VACT-CP group received 7 online modules provided as weekly sessions featuring an ECA named Coach Anne as their treatment guide. A detailed description of the VACT-CP program is available elsewhere [35]. The VACT-CP online intervention is based on manualized ACT for chronic pain treatments and workbooks [41-43]. ACT directly focuses on functioning and effective living in valued areas by increasing one’s psychological flexibility, defined as the ability to stay in contact with the present moment regardless of unpleasant thoughts, feelings, and bodily sensations, while choosing one’s behaviors based on the context and personal values. Psychological flexibility in ACT is composed of 6 major focal areas (ie, acceptance, cognitive defusion, being present, self as context, values, and committed action) that provide a framework for patients to re-engage with the present, their values, and their life choices [17]. By targeting pain-catastrophizing thought patterns and affective experiences (eg, negative emotions and urges to use illicit pain management substances) related to pain and pain avoidance, the aim of VACT-CP is to teach the skills necessary to engage in valued behaviors and functional improvement even while experiencing pain symptoms and comorbid mental health concerns.

All content in the VACT-CP online intervention was presented via simulated face-to-face conversations with the animated virtual guide Coach Anne. The 3D ECA, or “virtual coach,” appeared on the webpage and talked to the participant using synthetic speech and synchronized animated nonverbal behaviors, including hand gestures and facial displays. The participant could respond to Coach Anne’s questions using forced-choice text options and open-ended typed text that would trigger different responses from Coach Anne as the conversation progressed to allow the system to responsively interact in a personalized manner with the participant. Each of the 7 modules took approximately 15 to 20 minutes to complete, and additional resources such as mindfulness and guided imagery exercises were available at all times for participant use. Participants were sent a weekly email for the 7 weeks of VACT-CP involvement to remind them to complete each weekly module. They were not provided with a list of additional usual treatment options for pain available to them within the VHA.

#### WL+TAU Group

Veterans randomized to the WL+TAU group completed the same baseline session and all surveys at the same time points as those in the VACT-CP group. The term “waitlist” refers to participants being placed on a waitlist to use the intervention website, which was made available to them after their study participation (at week 11); “treatment as usual” refers to giving participants the option to engage in other traditional, common, and readily available treatment options relative to the treatment of their chronic pain condition. In the case of treatment as usual for chronic pain within this trial, participants were told to continue any ongoing treatment and were provided with a handout of additional common pain treatment options and resources available at the VA Bedford Healthcare System, in line with veteran-centered VHA pain treatment practices. Participants in the WL+TAU group were provided with additional referrals to these options as requested, and they were also encouraged to seek out any of the listed common pain treatment options over the course of the study. At the end of 11 weeks (final assessment), participants were provided with the VACT-CP online program URL and a staff-generated username and password so they could use the online VACT-CP system, if desired. There was no requirement that these participants must use the VACT-CP program. They were, however, informed that if they had any log-in or usability issues while using the website, they could contact the study staff for assistance.

### Outcomes

#### Demographics

Demographic measures included veterans’ self-reported information on survey questions related to pain type and duration, current age, gender (ie, man; woman; transgender man; transgender woman; genderqueer or gender nonconforming; and other, please specify), race (eg, White; Black or African American; American Indian or Alaska Native; Asian; and other, please specify), ethnicity (eg, Hispanic or Latino), and education level. This information was used to describe the demographics and health-related characteristics of the sample.

#### Feasibility, Usability, and Acceptability Measures

Measures of study feasibility, website usability, and intervention acceptability were assessed from baseline (week 0) to the primary final assessment (week 7) time points. Study feasibility was assessed using participant data (tracking of survey completion data, enrollment, retention, and attrition); usability was primarily assessed using the System Usability Scale (SUS) [44], which assesses the usability and learnability of technology through participant report. Veterans were asked to rate 10 statements on a 5-point Likert scale (1=strongly disagree to 5=strongly agree). All SUS items focused on their experience using the online chronic pain website, for example, “I would imagine that most people would learn to use this system very quickly.” Each item is rescored, totaled, and multiplied by 2.5 to get a total score ranging from 0 to 100. Higher scores indicate greater usability of a technology system***,*** with above***-***average usability of a system suggested by a score ***>***68, while a score ***>***80 indicates high usability. High internal reliability has been found in prior studies using the SUS (Cronbach α=0.91) [45], and the measure has also been successfully used in digital self-management interventions for chronic pain [46]. For this study, cutoff SUS scores that would indicate acceptable usability were set a priori to 68 [47]. Internal reliability for the SUS for this study was satisfactory (Cronbach α=0.78).

Acceptance of the VACT-CP website intervention was assessed using the Client Satisfaction Questionnaire-8 (CSQ-8) [48], which measures satisfaction with treatment services in an 8-item self-report survey. Items include questions on both quality and effectiveness (eg, “Have the services you received helped you to deal more effectively with your problems?”), with each statement rated on a 4-point Likert scale, ranging from 1 (low satisfaction) to 4 (high satisfaction), reflecting satisfaction with care. Items are summed for a total score ranging from 8 to 32, with higher scores indicating higher satisfaction with the treatment. Internal consistency for this sample was found to be acceptable for this measure (Cronbach α=0.93). In addition, intervention acceptance was evaluated using (1) the number of completed VACT-CP modules and (2) thematic coding of an open-ended item (“Is there anything additional you would like to tell us about the Veteran ACT for Chronic Pain website?”) asked on both the week-7 and 1-month follow-up surveys.

#### Pain Measures

Self-reports of pain severity and pain-related functioning were assessed using the Pain Outcomes Questionnaire-For Veterans [49], a 19-item inventory that assesses pain treatment outcomes in veteran populations. This self-report measure includes 5 domains: pain severity, mobility, vitality, activities of daily living, negative affect, and fear of activity. Each item is rated on a scale from 0 (never) to 10 (always), allowing for the calculation of domain scores by summing the ratings or determining a total score by averaging them. Final scores range from 0 to 10***,*** and higher scores represent greater impairment from pain for both domain scores and total scores. Within this study, the activities of daily living and pain severity domains were used, with pain functioning measured by the Pain Functioning subscale score of the Pain Outcomes Questionnaire (POQ) and the single-POQ Pain Severity item score used for assessing pain severity. This study sample’s internal reliability on the POQ Pain Functioning subscale was high (Cronbach α=0.90).

#### ACT Process Measures

Measures assessing major ACT processes related to chronic pain treatment (valued living, pain acceptance, and experiential avoidance) were assessed using the Chronic Pain Values Inventory (CPVI) [50], the Chronic Pain Acceptance Questionnaire (CPAQ) [51], and the Multidimensional Experiential Avoidance Questionnaire (MEAQ) [52], respectively.

The CPVI is a 12-item measure that assesses the relative importance of 6 value domains, perceived success in achieving those values, and the discrepancy between one’s importance of the value and their success at living in accordance with the value. This yields 3 distinct scores: CPVI-importance, CPVI-success, and CPVI-discrepancy (CPVI-D). Veterans were asked to rank the 6 value domains of work, health, family, friends, growth or learning, and intimate relations in order of importance to them. Importance was ranked on a 0 to 5 scale (from not at all important to extremely important). Following this, veterans rated how successful they felt in living their life in accordance with these values. Success and importance were rated using a 0 to 5 scale, with 0 being not at all successful and 5 being extremely successful. Mean scores were used to calculate the average success a veteran feels in pursuing values. Discrepancy scores were then calculated by averaging the differences between success and importance scores for each domain, with lower scores suggesting lower discrepancy between reported importance and success in valued living. For this study, the discrepancy scale (CPVI-D) was used to measure success in engagement in valued activities in the context of their perceived importance, with adequate reliability in this sample (Cronbach α=0.82).

The CPAQ measures the extent to which an individual can accept and move toward valued living, even when experiencing pain. This 20-item self-report questionnaire rates statements (eg, “I lead a full life even though I have chronic pain.”) on a 0 (never true) to 6 (always true) scale. The CPAQ yields a total score (CPAQ-total) from 0 to 120; it has 2 subscales: CPAQ–Activity Engagement and CPAQ–Pain Willingness [53]. Activity engagement reflects engagement in day-to-day activities despite the presence of pain, while pain willingness encompasses how willing someone is to engage in behavior that may result in pain and associated negative internal experiences. In previous research, adequate internal reliability has been demonstrated for the total score (Cronbach α=0.78) as well as for the activity engagement (Cronbach α=0.82) and pain willingness (Cronbach α=0.78) subscales, with higher scores on these subscales being associated with better emotional, social, and physical functioning [51]. For this study, internal reliability was high for the total score (Cronbach α=0.87) as well as the activity engagement (Cronbach α=0.87) and pain willingness (Cronbach α=0.86) subscales.

Finally, the MEAQ assesses experiential avoidance through 62 self-report items. Items are rated by the participant on a 1- to 6-point Likert scale (strongly disagree to agree). The MEAQ includes 6 distinct subscales: behavioral avoidance, distress aversion, procrastination, distraction and suppression, repression and denial, and distress endurance. This study used the behavioral avoidance subscale (example item: “I won’t do something if I think it will make me uncomfortable”)***,*** which has demonstrated good reliability (Cronbach α=0.81***-***0.90) in the extant literature [52,54] as well as within this study (Cronbach α=0.90).

#### Mental Health and Functioning Measures

Additional outcomes assessed the symptoms of common mental health comorbidities (ie, posttraumatic stress disorder [PTSD] and depression), mental health functioning, and physical health functioning. The Posttraumatic Stress Disorder Checklist [55] is a 20-item self-report measure that assesses PTSD symptoms across the past 30 days consistent with *DSM-5* criteria (eg, “Feeling very upset when something reminded you of the stressful experience”). Veterans were asked to rate how much each symptom bothered them on a 5-point Likert scale (0=not at all and 4=extremely). Analyses use total scores ranging from 0 to 80. The Posttraumatic Stress Disorder Checklist is a well-validated measure in veteran populations, demonstrating good internal reliability, test-retest reliability, and convergent or discriminant validity [56], and it has demonstrated usefulness in identifying provisional PTSD diagnoses and quantifying symptom severity [57]. In addition, the Patient Health Questionnaire-9 [58] assesses depressive symptoms across the 9 diagnostic criteria for major depressive disorder in the *DSM-IV* (example item: “Feeling, down, depressed or hopeless”). Frequency of depressive symptoms were assessed over the past 2 weeks using a 0 to 3-point Likert scale, with 0 representing “not at all” and 3 representing “nearly every day.” Total scores range from 0 to 27. The Patient Health questionnaire-9is a widely used measure within veteran populations across settings and has demonstrated usefulness in consideration of subthreshold major depressive disorder and common comorbidities [59].

To explore potential changes in functioning, participants completed the Veterans RAND 36-Item Health Survey (VR-36) [60], a 36-item self-report measure of health and related functioning across 8 domains: physical functioning, role limitations due to physical health, role limitations due to emotional problems, energy or fatigue, emotional well-being, social functioning, pain, and general health. The scale assesses the overall frequency or degree of health behaviors and impairment and health perceptions over time. For example, veterans were asked to rate the statement “How much bodily pain have you had during the past 4 weeks?” on a 6-point Likert scale, ranging from “none” to “very severe.” These 8 scales are summarized into physical (VR-36–Physical Component Score VR-36-PCS) and mental (VR-36–Mental Component Score VR-36-MCS) component summary scores.

### Sample Size

Consistent with the recommended stage model for development of behavioral therapies, the aim of this stage IB pilot feasibility RCT was to inform treatment development and future evaluation. To have 80% power to detect a medium effect size at a 2-tailed α level of 0.05, similar to past ACT intervention research studies, would require 125 participants, which is beyond the objective and scope of this preliminary pilot feasibility RCT. A total sample size of 40 (n=20 per group) is consistent with the recommendation by Rounsaville et al [61] of including 15 to 30 participants per condition or group for stage IB behavioral treatment development pilot testing, and the total goal for enrollment and randomization was 40 participants (n=20 per group) for the 7-week VACT-CP group and WL+TAU control group. Assuming an attrition rate of 30% after randomization based on past attrition rates for technology-delivered behavioral interventions [62], we planned to enroll up to 60 participants to meet our minimum goal of 40 successfully randomized participants over a projected 15 months.

### Randomization and Blinding

Randomization of individuals occurred immediately after enrollment in the study (ie, completion of the informed consent process and eligibility confirmation procedures). Participants were randomized 1:1 in 5 randomly permuted blocks of 10 to either the VACT-CP group or the WL+TAU comparison group to randomize participants into groups that resulted in equal sample sizes and ensure a balance in sample size across groups over time. Using Random.Org software (Random.Org LLC), the study PI (EDR) randomized participants to either group; participants were then informed of their group allocation in an encrypted email, followed by a phone call to confirm they understood their group. After allocation, participants received assessment information from a blinded research associate who was not aware of which group an individual was assigned to. Five total blocks were created for the study to account for potential randomization failures such as enrolled participants being lost to follow-up or the potential for additional veterans to be enrolled beyond the minimum goal of 40 participants.

### Statistical Methods

To address our a priori primary outcomes, we evaluated and described feasibility (rate of recruitment, retention, attrition, and intervention completion) and VACT-CP usability (SUS) and acceptability (CSQ-8***;*** program feedback and suggestions). Feasibility metrics included evaluating our ability to screen and enroll up to 60 participants over 15 months and obtain the desired final 40 eligible, randomized participants. We specifically aimed to enroll 2 to 3 participants per month. We projected a probable 30% attrition rate after randomization, resulting in an estimated sample of 28 participants who completed the study and approximately equivalent retention across the 2 groups. Acceptability and feedback were also evaluated using the open-ended survey item at week 7 and week 11, with participant feedback coded using the following 6 phases of thematic analysis [63]: reviewing the qualitative data (comments), generating initial codes, searching for themes, reviewing themes, defining and naming themes, and producing the final results using the constructed themes from common trends emerging from participant comments. Finally, we assessed feasibility and acceptability of the intervention by measuring the proportion of individuals who successfully completed the 7-week trial. Intervention completion was operationalized as completing 5 out of the 7 modules, which would be 63% of the program completed and 5 weeks of ACT intervention content. This is consistent with multiple online ACT pain interventions having between 3 and 8 modules or intervention weeks [62] as well as the mean percentage of completed sessions in previous online behavioral interventions [30].

As a secondary aim, we described pain severity, pain-related interference in daily functioning, pain acceptance, valued living, behavioral avoidance, and mental and physical functioning at baseline and follow-up (week 7) in each group and explored whether these scores changed from pre- to postintervention in each treatment group using Wilcoxon signed rank tests and outcome score categorization (eg, decrease, increase, *or* no change). Nonparametric tests were planned a priori due to the small sample size, which increased concerns that some outcome metrics would not be normally distributed. Secondary outcomes were analyzed using an intention-to-treat (ITT) approach, which included all randomized participants. To account for missing data, we used a “last observation called forward” method so that we could use the last completed follow-up as outcome data for all successfully randomized participants (n=3 using baseline data, n=2 using week 3 data, and n=37 using week 7 data). Analyses were conducted using SPSS Statistics (version 27; IBM Corp).

## Results

### Participant Flow and Baseline Data

**Figure 1:**
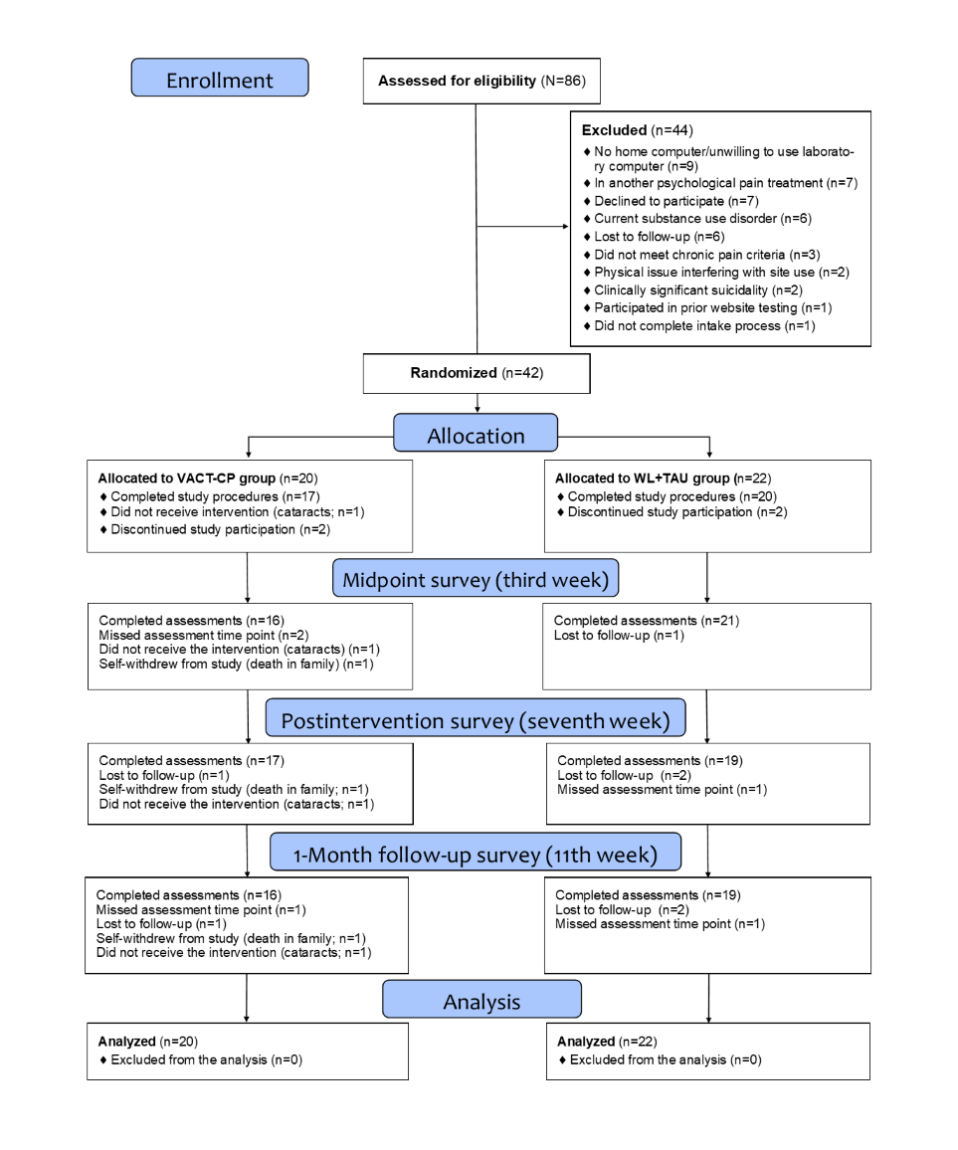
CONSORT (Consolidated Standards of Reporting Trials) flow diagram of participant inclusion and attrition. VACT-CP: Veteran Acceptance and Commitment Therapy for Chronic Pain; WL+TAU: waitlist plus treatment as usual.

The mean age of the randomized sample (n=42) was 53.7 (SD 15.2) years, with an average baseline NRS pain score of 7.1 (SD 1.7) out of 10, indicating moderately strong pain severity. Most identified as men (34/42, 81%) and White (36/42, 86%), with 10% (4/42) reporting Hispanic or Latino ethnicity. Most participants (38/42, 91%) had applied for and received VA disability compensation and had a VA service-connected disability rating (37/42, 88%). Of those participants, 11% (4/37) had a service-connected disability rating between 10% and 20%, 8% (3/37) had a rating between 30% and 40%, 11% (4/37) had a rating between 50% and 60%, 41% (15/37) had a rating between 60% and 90%, and 30% (11/37) had a 100% rating. Table 1 shows detailed demographic information and Figure 1 for participant flow. In addition, nonparametric independent-samples Mann-Whitney *U* tests were conducted to assess for imbalance between groups in baseline variables with only 2 variables. Across all secondary outcomes, 2 (CPAQ-total and the CPAQ–Activity Engagement) were significantly different between groups, with individuals in the VACT-CP group significantly lower at baseline on both measures (Table 2).

**Table 1:** Baseline characteristics of veterans with chronic pain, overall and by treatment group.

			Total participants (N=42)		VACT-CP^a^ group (n=20)		WL+TAU^b^ group (n=22)
Age (y), mean (SD)			53.7 (15.2)		50.6 (16.6)		56.5 (13.6)
Baseline pain NRS^c^, mean (SD)			7.1 (1.7)		7.2 (1.7)		7.0 (1.7)
Gender, n (%)							
	Men	34 (81)		15 (75)		19 (86)	
	Women	7 (17)		5 (25)		2 (14)	
	Transgender woman	1 (2)		0 (0)		1 (5)	
Race, n (%)							
	Asian American or Pacific Islander	1 (2)		1 (5)		0 (0)	
	Black or African American	1 (2)		1 (5)		0 (0)	
	White	36 (86)		16 (80)		20 (91)	
	Multiracial	4 (10)		2 (10)		2 (9)	
Ethnicity, n (%)							
	Hispanic or Latino	4 (10)		4 (20)		0 (0)	
	Not Hispanic or Latino	38 (90)		16 (80)		22 (100)	
Education level, n (%)							
	High school diploma or General Educational Development	2 (5)		0 (0)		2 (9)	
	Some college, no degree	15 (36)		6 (30)		9 (41)	
	Associate’s degree	5 (12)		3 (15)		2 (9)	
	Bachelor’s degree	10 (24)		6 (30)		4 (18)	
	Master’s degree	8 (19)		4 (20)		4 (18)	
	Doctoral degree	2 (2)		1 (5)		1 (5)	

^a^VACT-CP: Veteran Acceptance and Commitment Therapy for Chronic Pain.

^b^WL+TAU: waitlist plus treatment as usual.

^c^NRS: numeric rating scale.

**Table 2:** Baseline data differences in secondary outcomes by treatment group.

			VACT-CP^a^ group (n=20), median (IQR)		WL+TAU^b^ group (n=22), median (IQR)		*P* value
Pain measures							
	POQ-VA pain severity^c^	7.5 (6.0-8.8)		7.0 (6.0-8.0)		.70	
	POQ-VA pain functioning^d^	93.0 (76.0-108.5)		88.0 (80.1-101.7)		.50	
ACT^e^ process measures							
	CPVI-D^f^	1.8 (1.0-2.5)		1.8 (0.8-2.7)		.64	
	CPAQ^g^-total	66.0 (58.0-81.8)		80.0 (69.3-91.0)		.03	
	CPAQ-AE^h^	27.5 (22.0-37.0)		39.5 (28.5-45.0)		.02	
	CPAQ-PW^i^	38.5 (35.3-44.0)		44.5 (37.0-53.3)		.14	
	MEAQ-BA^j^	36.5 (26.5-45.0)		32.0 (24.0-42.3)		.49	
Mental health and functioning measures							
	PCL-5^k^	27.5 (17.5-42.0)		21.0 (9.0-35.3)		.24	
	PHQ-9^l^	18.5 (15.0-26.5)		17.0 (13.8-24.0)		.30	
	VR-36 PCS^m^	39.2 (30.8-48.9)		53.7 (32.9-65.6)		.32	
	VR-36 MCS^n^	44.6 (36.3-61.9)		52.5 (47.1-65.4)		.16	

^a^VACT-CP: Veteran Acceptance and Commitment Therapy for Chronic Pain.

^b^WL+TAU: waitlist plus treatment as usual.

^c^POQ-VA pain severity: Pain Outcomes Questionnaire–Short form–Pain Severity Score.

^d^POQ-VA pain functioning: Pain Outcomes Questionnaire–Short form–Pain Functioning Score.

^e^ACT: acceptance and commitment therapy.

^f^CPVI-D: Chronic Pain Values Inventory–Discrepancy Score.

^g^CPAQ-total: Chronic Pain Acceptance Questionnaire–Revised–Total Score.

^h^CPAQ-AE: Chronic Pain Acceptance Questionnaire–Activity Engagement.

^i^CPAQ-PW: Chronic Pain Acceptance Questionnaire–Pain Willingness.

^j^MEAQ-BA: Multidimensional Experiential Avoidance Questionnaire–Behavioral Avoidance Score.

^k^PCL-5: Posttraumatic Stress Disorder Checklist.

^l^PHQ-9: Patient Health Questionnaire-9.

^m^VR-36 PCS: Veterans RAND 36–Physical Component Score.

^n^VR-36 MCS: Veterans RAND 36–Mental Component Score.

### Primary Analyses: Feasibility, Usability, and Acceptability Outcomes

The rate of monthly recruitment was higher than expected, with approximately 4 to 5 veterans per month showing both initial interest and meeting all eligibility criteria for the study before randomization (n=44). This enabled recruitment to be completed in 10 months (April 2022 to February 2023) instead of the projected 15 months. Of the veterans who reported interest, 51% (44/86) met the initial study criteria via the combined telephone and medical record review screening and were scheduled for a baseline study appointment to confirm that inclusion criteria were met. During the baseline appointment, 2 (4%) of the 46 veterans declined to participate after reviewing the study information sheet due to (1) concerns that study tasks would take too much time and (2) concerns about being able to focus solely on the intervention content for pain management due to their occupational background (technology or user experience testing). Baseline procedures were successfully completed for the remaining 44 veterans, and we successfully randomized nearly all participants who consented (42/44, 95%), with 2 participants not randomized due to meeting the exclusion criteria on the SCID-5 related to substance use issues (n=1) and not completing the baseline survey (n=1).

The total rate of study attrition was 12% (5/42), with 15% (3/20) attrition in the VACT-CP group and 10% (2/22) in the WL+TAU group. Survey assessment completion was high across all time points, with completion rates of 88% (37/42) for the midpoint or week 3 survey, 88% (37/42) for the postintervention or week 7 survey, and 86% (36/42) for the 1-month follow-up or week 11 assessment. The average VACT-CP score on the SUS was 79.6 (SD 12.8), with a median score of 82.5 (IQR 70-87.5), indicating high usability. Most participants (15/17, 88%) reported a SUS score >68 (ie, acceptable usability cutoff score) and 65% (11/17) reported a score of ≥80 (ie, high usability cutoff score). In the VACT-CP group, 3 participants withdrew after randomization, with 1 withdrawing before accessing the website due to an increase in cataract symptoms that precluded use of the website. Across the 19 total website users, the mean number of completed modules was 5 (SD 2.14) out of 7 total in the weekly program (median 7); 63% (12/19) of the participants completed at least 5 modules, the a priori set minimum number of completed modules for intervention dosage. Table 3 shows detailed module completion rates.

Participants’ average satisfaction scores for the overall VACT-CP program were high (mean 23.7, SD 5.2; median 24, IQR 8-32), with 82% (14/17) of participants rating the overall quality of the VACT-CP program as good or excellent on that CSQ-8 item at week 7. During the rapid thematic analysis of the open-ended survey items from the week 7 and week 11 surveys, negative and positive feedback themes were identified. For the only 3 participants who rated the overall program quality as fair or poor, information from the website feedback survey item provided more information on user perceptions. These 3 participants cited issues with usability related to the speed, awareness of the ECA as nonhuman, and 1 content-related suggestion to better separate physical and mental health concerns in the program. Positive comments from users included perceived program tailoring, acceptability of ACT content, perceived usefulness of the program for pain management, appropriateness of VACT-CP for a military population, and high perceived ease of use. No serious adverse events or unintended effects were reported from either group in exit interviews or during phone check-ins. Table 4 shows representative quotes across each theme, paired with information on the participant’s number of modules completed, SUS total score, and CSQ-8 total score (module range 0-7, CSQ-8 range 8-32, and SUS range 0-100.).

**Table 3:** Completion of modules among veterans with chronic pain randomized to the VACT-CP^a^ group (N=19).

Number of VACT-CP modules	Number of participant completers, n (%)
0 completed modules	0 (0)
1 completed module	0 (0)
2 completed modules	4 (21)
3 completed modules	1 (5)
4 completed modules	2 (11)
5 completed modules	0 (0)
6 completed modules	2 (11)
7 completed modules	10 (53)

^a^VACT-CP: Veteran Acceptance and Commitment Therapy for Chronic Pain.

**Table 4:** Thematic map of Veteran Acceptance and Commitment Therapy for Chronic Pain (VACT-CP) feedback and recommendations.

Theme			Representative quote		
Positive feedback					
	Program tailoring	“The encouraging remarks were great. I loved the ability to set my own goals and the interactions between my set goals and the other established goals coach Anna [sic] had for me.” [Number of modules completed=4, Client Satisfaction questionnaire-8 (CSQ-8) score=25, System Usability Scale (SUS)=83]			
	Acceptability of acceptance and commitment therapy (ACT) content	“I loved the meditation help from the program. I think others will enjoy it to [sic].” [Number of modules completed=7, CSQ-8 score=31, SUS=85] “I liked being able to identify a thought as just a thought.” [Number of modules completed=6, CSQ-8 score=23, SUS=85]			
	Appropriateness to a military population	“It was a great idea and should be continued for more active and retired Vets.” [Number of modules completed=7, CSQ-8 score=31, SUS=85]			
	Perceived usefulness and impact on pain management	“I found it helped me accept my pain where I used to blame myself for it.” [Number of modules completed=7, CSQ-8 score=30, SUS=100] “It has definitely helped to change my mindset on my pain. The different outlooks and methods to calm my pain were very helpful in maintaining healthy relationships with my family.” [Number of modules completed=4, CSQ-8 score=25, SUS=83]			
	High usability	“Overall worked very well and was very easy to use.” [Number of modules completed=4, CSQ-8 score=25, SUS=83] “I think it was well done and...I am hard put to find anything that could have been made better with the website. The idea behind the website seems to be well thought out. I would recommend this website to almost anyone.” [Number of modules completed=7, CSQ-8 score=30, SUS=98]			
Negative feedback					
	Usability concerns	“I’m sorry to say that because it has a lot of really good information, but I really hate how slow the speed is.... The content itself is fine but I wish I could skip and read it.... I think my study login got messed up too, multiple times I’d log in and it’d be weeks behind, like today I logged in and I could see up to module 7 but then I got kicked out and only saw up to module 3.” [Number of modules completed=2, CSQ-8 score=10, SUS=50]			
	Therapeutic content issues	“I recommend separating mental health and chronic physical pain.” [Number of modules completed=3, CSQ-8 score=16, SUS=68] “There should be more interacting at the end of the session. Something to help ground a person after bringing up old memories. Like good news story totally unrelated to the session and asking your opinion of it. Or what 5 things do you notice around you. Just something to ground us PTSD people.” [Number of modules completed=7, CSQ-8 score=22, SUS=88] “I think a combination between website and human interaction to better understand how to implement the content would be helpful.” [Number of modules completed=6, CSQ-8 score=22, SUS=80]			
	Feedback on the embodied conversational agent Coach Anne	“Sometimes Anne was difficult to understand [the way she spoke/pronounced words/annunciated] before I got used to the program.” [Number of modules completed=7, CSQ-8 score=24, SUS=70] “I’m aware that coach Ann [sic] isn’t a real person. So, the previous questions about her respecting or liking me are silly. However, her edges are on point!” [Number of modules completed=7, CSQ-8 score=23, SUS=70]			

### Secondary Analyses: Pain, Functioning, and ACT Process Measure Outcomes

There were no significant differences from baseline to the week 7 assessment in the WL+TAU group on pain severity, pain-related functioning, chronic pain values discrepancy, chronic pain acceptance, active engagement, pain willingness, behavioral avoidance, PTSD symptom severity, depression symptom severity, mental health functioning, or physical health functioning (Table 5). There were also no significant differences from pre- to postintervention for the VACT-CP group on pain severity, pain-related functioning, chronic pain values discrepancy, pain willingness, behavioral avoidance, mental health functioning, and physical health functioning (Table 5).

However, in the VACT-CP group only, there was a statistically significant difference for participants on their chronic pain acceptance total scores (*z* score=–3.48; *P*<.001) from pre- (median 66.0, IQR 58.0-81.8) to postintervention (median 80.0, IQR 67.3-85.5), with 80% (16/20) of the participants reporting an increase in pain acceptance, 15% (3/20) reporting no change, and 5% (1/20) reporting a decrease in pain acceptance following the use of the online intervention. There was also a significant improvement in active engagement scores in the VACT-CP group only, (*z* score=–3.20; *P*=.001) from pre- (median 27.5, IQR 22.0-37.0) to postintervention (median 36.0, IQR 26.7-42.5), with 80% (16/20) of the participants reporting increased active engagement, 15% (3/20) reporting no change, and 5% (1/20) reporting decreased active engagement (ie, the degree to which one engages in valued life activities regardless of pain). Finally, there was a significant improvement in depression symptom severity scores in the VACT-CP group only, (*z* score=–2.14; *P*=.03) from pre- (median 18.5, IQR 15.0-26.5) to postintervention (median 18.00, IQR 13.0-24.8), with 60% (12/20) of the participants reporting a decrease in depressive symptoms, 20% (4/20) reporting no change, and 20% (4/20) reporting an increase in depressive symptoms. Table 5 shows specific secondary outcome estimates.

**Table 5:** Changes in secondary outcomes from baseline to postintervention (week 7 assessment) by treatment group.

			VACT-CP^a^ group (n=20)							WL+TAU^b^ group (n=22)				
			Baseline, median (IQR)		Postintervention or week 7, median (IQR)		*P* value		Baseline, median (IQR)			Postintervention or week 7, median (IQR)		*P* value
Pain measures														
	POQ-VA pain severity^c^	7.5 (6.0-8.8)		7.0 (5.3-9.0)		.60		7.0 (6.0-8.0)			7.5 (5.0-8.3)		.75	
	POQ-VA pain functioning^d^	93.0 (76.0-108.5)		92.5 (70.5-108.8)		.59		88.0 (80.1-101.7)			83.0 (77.8-99.6)		.54	
ACT^e^ process measures														
	CPVI-D^f^	1.8 (1.0-2.5)		1.5 (0.7-2.6)		.30		1.8 (0.8-2.7)			1.8 (0.8-2.4)		.64	
	CPAQ^g^-total	66.0 (58.0-81.8)		80.0 (67.3-85.5)		<.001		80.0 (69.3-91.0)			82.0 (70.50-90.0)		.78	
	CPAQ-AE^h^	27.5 (22.0-37.0)		36.0 (26.7-42.5)		.001		39.5 (28.5-45.0)			36.5 (32.8-44.0)		.79	
	CPAQ-PW^i^	38.5 (35.3-44.0)		41.5 (36.5-45.0)		.19		44.5 (37.0-53.3)			45.5 (35.8-50.2)		.54	
	MEAQ-BA^j^	36.5 (26.5-45.0)		39.5 (36.0-44.5)		.07		32.0 (24.0-42.3)			32.5 (27.3-42.3)		.13	
Mental health and functioning measures														
	PCL-5^k^	27.5 (17.5-42.0)		31.0 (12.8-40.8)		.63		21.0 (9.0-35.3)			21 (10.5-29.3)		.65	
	PHQ-9^l^	18.5 (15.0-26.5)		18.0 (13.0-24.8)		.03		17.0 (13.8-24.0)			18.5 (15.0-23.0)		.40	
	VR-36 PCS^m^	39.2 (30.8-48.9)		41.3 (27.7-52.1)		.68		53.7 (32.9-65.6)			52.6 (34.9-66.7)		.38	
	VR-36 MCS^n^	44.6 (36.3-61.9)		46.4 (30.4-61.2)		.82		52.5 (47.1-65.4)			53.4 (42.4-65.8)		.76	

^a^VACT-CP: Veteran Acceptance and Commitment Therapy for Chronic Pain.

^b^WL+TAU: waitlist plus treatment as usual.

^c^POQ-VA pain severity: Pain Outcomes Questionnaire—Short form—Pain Severity Score.

^d^POQ-VA pain functioning: Pain Outcomes Questionnaire—Short form—Pain Functioning Score.

^e^ACT: acceptance and commitment therapy.

^f^CPVI-D: Chronic Pain Values Inventory—Discrepancy Score.

^g^CPAQ-Total: Chronic Pain Acceptance Questionnaire–Revised—Total Score.

^h^CPAQ-AE: Chronic Pain Acceptance Questionnaire–Activity Engagement.

^i^CPAQ-PW: Chronic Pain Acceptance Questionnaire–Pain Willingness.

^j^MEAQ-BA: Multidimensional Experiential Avoidance Questionnaire—Behavioral Avoidance Score.

^k^PCL-5: Posttraumatic Stress Disorder Checklist.

^l^PHQ-9: Patient Health Questionnaire-9.

^m^VR-36 PCS: Veterans RAND 36—Physical Component Score.

^n^VR-36 MCS: Veterans RAND 36—Mental Component Score.

### Additional Post Hoc Analyses by Intervention Dosage

We conducted secondary exploratory analyses to better understand the relationship between secondary outcomes by the “dosage” received of the VACT-CP program. Given our predefined intervention completion “dose” of VACT-CP completion to be 5 out of 7 modules, we descriptively examined differences in the outcomes between those participants who received the predefined VACT-CP intervention dosage (ie, at least 5 of the 7 modules, n=12 participants versus participants who did not meet the VACT-CP intervention dosage criteria (ie, <5 modules, n=7 participants). There were no differences at baseline in secondary outcomes between those who received the predefined intervention dosage (n=12) versus those who did not (n=7). In terms of significant differences in outcomes from pre- to postintervention by VACT-CP completer group, completers did not report better outcomes than noncompleters on pain severity, pain-related functioning, chronic pain values discrepancy, pain willingness, behavioral avoidance, depression, PTSD symptoms, mental health functioning, and physical health functioning. Similar to the ITT analysis, completers of the VACT-CP program had a significant increase from baseline to postintervention in chronic pain acceptance total scores (*z* score=–2.65; *P*=.008) from pre- (median 67.7, IQR 52.5-84.5) to postintervention (median 78.2, IQR 67.8-89.0), as did those who did not fully complete VACT-CP (*z* score=–2.06; *P*=.03) from pre- (median 74.4, IQR 64.0-78.0) to postintervention (median 77.1, IQR 68.0-82.0). Similar to the results of the ITT analysis, completers of the VACT-CP program had a significant increase from baseline to postintervention in CPAQ active engagement total scores (*z* score=–2.49; *P*=.01) from pre- (median 29.9, IQR 22.0-37.0) to postintervention (median 36.5, IQR 27.5-44.0), as did users who did not fully complete the program (*z* score=–2.03; *P*=.04) from pre- (median 33.1, IQR 25.0-42.0) to postintervention (median 36.4, IQR 29.0-43.0).

## Discussion

### Primary Feasibility, Usability, and Acceptability Findings

This pilot feasibility RCT examined an online ACT intervention for chronic pain in veterans (VACT-CP) compared to a WL+TAU control group. The primary aim of the RCT was to evaluate the feasibility of the study design and method of VACT-CP delivery, and it revealed that both were feasible and acceptable. Despite the closure of many hospital clinics and the need to shift our original protocol to require that all study activities be completed remotely for at least a portion of the clinical trial due to the COVID-19 pandemic, we were able to recruit and randomize participants at nearly double the anticipated rate (4-5 vs a projected 2-3 participants per month), with recruitment completed in 66% of the time originally projected (in 10 months vs the originally anticipated 15 months), suggesting high veteran interest in online chronic pain programming. Retention rates in each group were also high, with similar attrition in each group. Finally, the rates of completion for the study assessments were also high, with completion rates >85% for all 4 assessment time points, suggesting high study protocol feasibility as well. Although some prior studies have suggested high rates of attrition for veterans in psychotherapy treatments [64], our study reported high retention. This is in line with a recent meta-analysis suggesting higher retention in virtual interventions and, in particular, those with acceptance-based interventions that offer participants monetary compensation for survey completion and remind participants to engage in the intervention [65]. Though the literature base for factors that affect veteran engagement in virtual care within VHA is robust, more work is needed to discover successful strategies to adopt and sustain digital product engagement to assist veteran functioning and quality of life [66].

Central to the purpose of our project was the expectation that the VACT-CP program would be found highly usable, acceptable, and useful by veterans with chronic pain. In terms of usability of the VACT-CP program, nearly all scores on the SUS were well above the minimum cutoff score of 68 (17/19, 89%), indicating that participants found the intervention website highly usable from their PCs. In addition to being highly usable, rates of sustained VACT-CP website use were satisfactory, with 63% (12/19) of the participants completing the predefined minimum dosage for intervention engagement with 6 (86%) of the 7 modules completed, a common metric for online pain self-management programs in similarly aged adult populations [46]. This rate is better than or comparable with past meta-analysis-reported completion rates for internet-delivered ACT programs of between 39% and 97% [30,67].

However, it is important to note that only 53% (10/19) of the participants completed all 7 weekly modules. The lower rate of full program completion may be explained in part by system difficulties that resulted in errors in the scheduled delivery of modules each week, which occurred during the final 3 months of the RCT and affected 5 users. Of these 5 users, follow-up phone calls revealed that 2 users clearly reported that they stopped using the website because they could not progress and 2 insisted that they completed all 7 modules, although the website data show that both only completed 6 modules and not the final seventh module. This may also be attributed to user confusion, as participants might have mistakenly believed that the anytime Mindfulness module was 1 of the 7 full program modules; or, this discrepancy could be related to a website data capture issue. As a conservative estimate, we used website use statistics to report on completion rates as opposed to self-reports. Given these usability issues and their impact on the acceptability and feasibility of delivering the full intervention, the website module tracking was fixed following this feedback. However, future efficacy and implementation trials in VACT-CP will attempt to improve full intervention adherence by including improved software design to guarantee timely delivery of each module weekly on the website as well as incorporating more than a single reminder each week when the next week’s module becomes available.

The acceptability and personalized tailoring of the ACT program content to individual interactions using the ECA, Coach Anne, was demonstrated through high treatment satisfaction scores on par with other internet-delivered chronic pain therapeutic interventions [29,68]. Furthermore, qualitative feedback showed that VACT-CP participants responded positively to the dialogue-based tailoring of website ACT content and that they found the program useful in learning new pain management techniques (eg, acceptance and mindfulness). At the same time, users had multiple recommendations to improve the program. For instance, 1 veteran reported that acceptability would be enhanced by increased personalization of content based on user preference (for instance, separating mental and physical aspects of pain content and adding more content for veterans struggling with substance use). This aligns with a growing emphasis on personalized digital health products in veteran care. Emerging research has shown a veteran preference for tailored digital products [69] and comfort with virtual agents in delivering health care programming [70]. In addition, VHA strategic priorities have emphasized interventions that increase access to veteran-centered and highly accessible treatment options [11]; this information is critically important to understanding acceptability and feasibility. While this excellent feedback has been beneficial to continued website revision plans, the overall results suggest that the original VACT-CP program delivered a highly acceptable, accessible, and well-guided pain self-management experience for veterans at home.

This sample was well informed about the study’s purpose, which was to evaluate the usability, acceptability, and feasibility of the online pain program. Participants were recruited not only to experience the intervention but also to contribute to potential quality improvements in the virtual technology. The sample of veterans, all of whom were VA patients, also reported higher education levels than the national averages for veterans, with 57% reporting an associate’s degree or higher level of education compared to the national rates of 42% among veterans [71]. This speaks to the importance of further investigating the digital divide in the veteran communities; previous research has shown that veterans who receive care in the VA report better digital health knowledge and skills compared to veterans who do not receive care within the VA [72]. This ability to use health-related technology has also been theorized to reflect veterans’ education levels [73]. Taken together, it is likely that the participants of this study were more active users of VACT-CP than the general veteran population, who were perhaps more skilled at technology use or even online educational programs. At the same time, given that this product is intended as a veteran-chosen, at-home digital option for pain self-management, it is likely that future users would be similar in terms of interest and digital literacy. Future studies should assess whether digital literacy and education levels predict intervention engagement and whether efforts to increase digital literacy in this population will result in more equitable access to online pain management programs.

### Secondary Pain, Mental Health, and ACT Process Outcomes

The primary purpose of this pilot feasibility RCT was to assess the feasibility, usability, and acceptability of the study protocol and online intervention, and our sample size was determined for this primary aim. Consequently, our secondary aim to describe changes in pain and mental and physical functioning had limited statistical power, drew from a particularly small sample to assess within-group differences, and should be interpreted as hypothesis generating rather than definitive. In terms of descriptive results, we observed increases in participants’ baseline to postintervention scores for mental and physical functioning as well as decreases in pain severity, pain-related interference in daily functioning, and discrepancy in valued living among participants randomized to the VACT-CP program, suggesting that future fully powered efficacy trials may show benefit for participants of our digital ACT intervention to help them manage their chronic pain.

At the same time, participants who received the VACT-CP intervention showed significant improvements on measures of pain acceptance and specifically increased activity engagement, while those in the WL+TAU group did not. These findings were true in both the ITT analysis and post hoc exploratory analyses based on intervention dosage, suggesting that even a smaller dose of the intervention could result in improvements in pain acceptance. These findings provide preliminary support that VACT-CP increases pain acceptance in the context of values-based action, the hypothesized mechanism of change in ACT interventions for chronic pain management (eg, [74,75]). In addition, depression scores for VACT-CP users, but not those in the WL+TAU group, significantly decreased following participation in this digital pain management intervention. This also aligns with research showing that ACT, as a transdiagnostic intervention, can concurrently address additional underlying issues related to both chronic pain and comorbid psychiatric symptoms [76]. Although these findings are promising, the small sample size inherent in this study and many other pilot studies is a major limitation, and consequently, care should be exercised in interpreting these secondary outcome analyses. The primary aim of this study was to evaluate the feasibility and acceptability of VACT-CP program and study procedures. A fully powered efficacy trial is needed to examine changes in functioning, mental health, and ACT outcomes.

### Limitations and Future Directions

This study has additional strengths and limitations. First, as the purpose of this study was to complete a preliminary evaluation of study procedures and the newly developed VACT-CP system, the inclusion criteria were specifically set to include a wide range of veterans with chronic pain while excluding those who might potentially require a higher level of care for comorbid mental health issues, including substance use issues. However, the rates of comorbidity for chronic pain and these conditions are quite prevalent in veteran populations as, in an effort to alleviate pain, many individuals turn to substances such as alcohol, tobacco, and opioids [77,78], leading to high rates of co-occurring chronic pain and SUDs [79]. Future research is needed to investigate whether VACT-CP, perhaps with additional content and tailoring, might be acceptable and useful to veterans with both chronic pain and comorbid addiction. Second, as the goal of this study was to examine feasibility, usability, and acceptability of the online intervention and study procedures, we did not have adequate statistical power to detect meaningful changes in secondary outcomes, and the results of these exploratory analyses should be viewed as preliminary. In addition, there were baseline differences between the 2 groups on chronic pain acceptance. Finally, it is not clear from this preliminary study how VACT-CP might compare to a more active control condition. Future research should include a control group that allows for matched time and attention as well as pain psychoeducation for more robust comparison testing and to allow for additional analysis of the proposed mechanism of change in VACT-CP (psychological flexibility).

### Conclusions

The findings of this pilot RCT suggest that the VACT-CP online intervention is a highly usable, acceptable, accessible, and engaging method for delivering tailored ACT for chronic pain to veterans. Given these positive preliminary findings, we believe that one of the main opportunities for this continued work is to further enhance veterans’ engagement with the online program in ways that attend to their individual comorbid health concerns and support their personalized goals. Findings from this pilot RCT indicate that the current VACT-CP platform warrants further scientific inquiry to investigate both the efficacy of an internet-based ACT self-help program for individuals with chronic pain. In addition, it is important to explore the underlying mechanisms (eg, acceptance, mindfulness, and value-based living) that may predict changes in functioning and quality of life. Given the impact of poorly self-managed chronic pain on nearly every aspect of functioning and quality of life, it is vital that highly accessible, usable, and effective digital products be provided to veterans as a pain treatment option.

## Appendix

Multimedia Appendix 1 CONSORT-eHEALTH checklist (V 1.6.1).

## Abbreviations

ACT: acceptance and commitment therapy
CPAQ: Chronic Pain Acceptance Questionnaire
CSQ-8: Client Satisfaction Questionnaire-8
DSM-5: Diagnostic and Statistical Manual of Mental Disorders, Fifth Edition
ECA: embodied conversational agent
ITT: intention-to-treat
MEAQ: Multidimensional Experiential Avoidance Questionnaire
PI: principal investigator
POQ: Pain Outcomes Questionnaire
PTSD: posttraumatic stress disorder
RCT: randomized controlled trial
SUD: substance use disorder
SUS: System Usability Scale
VA: Veterans Affairs
VACT-CP: Veteran Acceptance and Commitment Therapy for Chronic Pain
VHA: Veterans Health Administration
VR-36: Veterans RAND 36-Item Health Survey
VR-36-MCS: Veterans RAND 36-Item Health Survey–Mental Component Score
VR-36-PCS: Veterans RAND 36-Item Health Survey–Physical Component Score
WL+TAU: waitlist plus treatment as usual

## Data Availability

The datasets generated and analyzed during this study are not publicly available due to the security requirements of the Department of Veterans Affairs but are available from the corresponding author on reasonable request. The authors will consider reasonable requests on a case-by-case basis, subject to compliance with the Department of Veterans Affairs data sharing agreements.
